# In This Issue

**Published:** 2006

## AN EPIDEMIOLOGIC ANALYSIS OF CO-OCCURRING ALCOHOL AND TOBACCO USE AND DISORDERS

To better understand the scope of the adverse consequences of alcohol and tobacco use and to design more effective treatment and prevention strategies, it is important to have accurate and reliable information on the prevalence of alcohol and tobacco use and associated disorders. Many existing prevalence studies, however, are based on outdated data and diagnostic criteria. Therefore, the 2001–2002 National Epidemiologic Survey on Alcohol and Related Conditions (NESARC) sought to provide a current and statistically sound assessment of the prevalence of concurrent alcohol and tobacco use in the United States. According to Drs. Daniel E. Falk, Hsiao-ye Yi, and Susanne Hiller-Sturmhöfel, the survey data indicate that 27.5 percent of men and 16.4 percent of women used both alcohol and tobacco, and 4.1 percent of men and 1.8 percent of women had concurrent alcohol use disorders and nicotine dependence. The prevalence of alcohol and tobacco use depended on both age and ethnic group. These results suggest that some population subgroups may be at particular risk for problems from drinking and smoking. Identifying these high-risk groups, such as younger men and Native Americans/Alaskan Natives, can help health care policymakers and treatment planners create more focused treatment and prevention strategies. (pp. 162–171)

## CO-OCCURRING RISK FACTORS FOR ALCOHOL DEPENDENCE AND HABITUAL SMOKING

Epidemiologic studies have shown that people who are dependent on alcohol are three times more likely to also be dependent on nicotine, and people who are dependent on nicotine are four times more likely to be dependent on alcohol compared with the general population. Genetic factors play a significant role in both alcohol and nicotine dependence, and studies in twins have shown that people with family histories of alcohol and nicotine dependence are 50 to 60 percent more likely to develop problems with alcohol and smoking. In this article, Drs. Richard A. Grucza and Laura J. Bierut review findings from the Collaborative Study on the Genetics of Alcoholism (COGA) that have identified several chromosomal regions and specific genes that may contribute to the development of alcohol dependence and habitual smoking. Although some of these genetic factors are specific to dependence on one drug, others are associated with dependence on both drugs. (pp. 172–178)

## ALCOHOL’S ACTIONS ON NEURONAL NICOTINIC ACETYLCHOLINE RECEPTORS

Because alcohol and nicotine often are used together, it is likely that they act through common mechanisms in the brain. In this article, Ms. Tiffany J. Davis and Dr. Christopher M. de Fiebre report that one of these mechanisms involves nicotinic acetylcholine receptors (nAChRs), which mediate the actions of nicotine. Researchers have demonstrated that alcohol acts on some nAChR subtypes but has little effect on others. In chronic smokers, alcohol may alter the effects associated with smoking by acting on nAChRs in the brain, reversing desensitization to nicotine. Additionally, both alcohol and nicotine can lead to changes in the number of some nAChRs; further research may show whether these changes are responsible for the development of cross-tolerance between alcohol and tobacco. Studies also suggest that natural variation in the genes that code for different subtypes of nAChRs may be associated with sensitivity to alcohol and to nicotine. Finally, the presence of some nAChR receptors may protect against alcohol’s toxic effects on the nervous system. (pp. 179–185)

## BIOLOGICAL PROCESSES UNDERLYING CO-USE OF ALCOHOL AND NICOTINE

Alcohol and nicotine often are used together despite the fact that their effects and mechanisms of action are quite different. Nicotine is classified as having stimulant effects and acts on the brain by directly binding to and activating a molecule called the nicotinic acetylcholine receptor. Alcohol, on the other hand, is classified as a depressant and does not directly bind to any one receptor site. In this article, Drs. Douglas Funk, Peter W. Marinelli, and Anh D. Lê describe the biological mechanisms that may contribute to the concurrent use of alcohol and nicotine. The authors present evidence that a certain brain signaling system—the mesolimbic dopamine system—participates in the interaction of alcohol and nicotine in the brain, mediating the reinforcing and rewarding effects of both drugs. Another biological factor that may contribute to co-occurring alcohol and nicotine use is cross-tolerance—that is, reduced sensitivity to one drug resulting from chronic use of another drug—which may result in increased use of either drug. Finally, the authors present evidence that genetic factors may predispose people to the co-use of alcohol and nicotine. (pp.186–192)

## CANCER RISK ASSOCIATED WITH ALCOHOL AND TOBACCO USE

Alcohol and tobacco, independently, take an enormous toll on public health; moreover, a growing body of evidence suggests that these substances might be especially harmful when they are used together. As Drs. Claudio Pelucchi, Silvano Gallus, Werner Garavello, Cristina Bosetti, and Carlo La Vecchia report, both alcohol and tobacco place users at risk for certain cancers, particularly those of the upper aero-digestive tract (i.e., the oral cavity, throat [pharynx], voice box [larynx], and esophagus), and risk of these cancers typically increases with the amount of alcohol or nicotine consumed. Furthermore, in many cases combined use of both drugs increases the effects on risk. Overall, combined use of alcohol and tobacco accounts for approximately 80 percent of oral and pharyngeal cancer cases in men and about 65 percent of cases in women. Similarly, more than 80 percent of esophageal cancer cases in Europe and the Americas can be attributed to combined alcohol and tobacco use. Some studies also have reported that alcohol and tobacco may work synergistically to increase the risk of liver cancers; however, more research is needed to explore this issue. (pp. 193–198)

## THE EFFECTS OF SMOKING AND DRINKING ON CARDIOVASCULAR DISEASE AND RISK FACTORS

Smoking and excessive alcohol consumption, independently, have been shown to have similar, often negative, effects on some forms of cardiovascular disease; however, several factors make this a complicated issue. In this article, Dr. Kenneth J. Mukamal examines how, although smoking increases the risk for all forms of cardiovascular disease, moderate drinking has been shown to decrease the risk for heart attacks, ischemic strokes, and congestive heart failure but increase the risk for hemorrhagic stroke. In addition, there is relatively little evidence that the effects are worse when smoking and drinking occur together than would be expected from their independent effects. (pp. 199–202)

## TOBACCO CESSATION TREATMENT FOR ALCOHOL-DEPENDENT SMOKERS: WHEN IS THE BEST TIME?

Many people with alcohol use disorders also smoke. In population-based studies of adults who abuse alcohol or who are alcohol dependent, 33 to 44 percent smoke, and in the population of adults seeking treatment from alcohol abuse and dependence, up to 80 percent are smokers. In this article, Drs. Molly Kodl, Steven S. Fu, and Anne M. Joseph review the evidence regarding the effects of smoking cessation treatment on alcohol treatment outcomes and the advantages of simultaneous tobacco treatment versus treating alcohol and nicotine dependencies independently. Although many smokers in alcohol treatment programs express the desire to quit smoking, the question of when this should be done is debatable. (pp. 203–207)

## SMOKING CESSATION AND ALCOHOL ABSTINENCE: WHAT DO THE DATA TELL US?

People who both drink and smoke heavily are more likely to die of complications from smoking than from alcohol. However, many alcoholism treatment programs—even those that address multiple addictions—are unlikely to address cigarette smoking. According to Drs. Suzy Bird Gulliver, Barbara W. Kamholz, and Amy W. Helstrom, this reluctance to treat both addictions results from widespread myths about alcohol and nicotine dependence, including the notion that quitting both alcohol and tobacco is too difficult for patients. However, the authors show that efforts to quit smoking may actually improve alcohol-related outcomes: according to the authors, smoking cessation intervention is associated with a 25 percent greater likelihood of abstinence from alcohol and other drugs in the long term. Reasons for this correlation remain largely unexplored but may include greater clinical contact time (patients receive more treatment because they are being treated for two disorders rather than one), reduced exposure to cues that trigger substance use, relapse prevention and/or coping skills practice, increased mastery or self-efficacy, and broader healthy lifestyle choices. (pp. 208–212)

## TREATING SMOKING DEPENDENCE IN DEPRESSED ALCOHOLICS

Compared with the general population, smokers with alcohol dependence or other psychiatric disorders have an increased risk of adverse health consequences, a higher mortality rate, and a lower treatment success rate. Research has shown that alcohol and nicotine cravings positively correlate with depression and anxiety, making it difficult to treat each condition separately. For example, people who are trying to stop using alcohol often turn to nicotine in response to the discomfort associated with the urge to drink and to improve their mood. In addition, alcohol and nicotine have been shown to interact with certain brain chemicals known as central opioid peptides, which induce pain relief and feelings of euphoria. In this article, Drs. Nassima Ait-Daoud, Wendy J. Lynch, J. Kim Penberthy, Alison B. Breland, Gabrielle Marzani-Nissen, and Bankole A. Johnson describe pharmacotherapeutic and psychotherapeutic options for treating co-occurring nicotine and alcohol dependence and psychiatric disorders and discuss the limitations of these treatments as well as introduce new approaches. (pp. 213–220)

## CIGARETTE SMOKING AMONG ADOLESCENTS WITH ALCOHOL AND OTHER DRUG USE PROBLEMS

Smoking is extremely common among youth diagnosed with alcohol and other drug (AOD) use disorders. However, studies of smoking cessation efforts in this population are rare. In this article, Drs. Mark G. Myers and John F. Kelly describe developmental differences between adolescents and adults that create special challenges in treating adolescent tobacco dependence. According to the authors, interventions that target youth must take into account special considerations, such as the importance of peer influences for adolescents and some adolescents’ lack of motivation to quit. (Adolescents, who are less likely than adults to experience tobacco-related health problems, also tend to be less motivated to quit smoking.) The authors suggest that client-centered, motivation-enhancing tobacco interventions, when modified with these considerations in mind—for example, by emphasizing group over individual interventions and educating patients about the effects of tobacco and nicotine—can be effective in helping adolescents quit smoking and are not detrimental to AOD-related treatment outcomes. (pp. 221–227)

## BARRIERS AND SOLUTIONS TO ADDRESSING TOBACCO DEPENDENCE IN ADDICTION TREATMENT PROGRAMS

Despite the high prevalence of smoking among people recovering from addiction, tobacco dependence is rarely addressed in addiction treatment programs. In this article, Drs. Douglas M. Ziedonis, Joseph Guydish, Jill Williams, Marc Steinberg, and Jonathan Foulds identify and address barriers to treating tobacco dependence among patients in addiction treatment programs. These barriers include the staff’s reluctance to participate and lack of training, “clinical lore” or misinformation about tobacco (such as the myth that quitting tobacco will worsen other substancerelated outcomes), and lack of resources. According to the authors, programs must be modified to recognize and treat tobacco dependence—for example, by screening for tobacco use, offering nicotine replacement therapies and smoking cessation medication on inpatient units, and bundling costs so that programs can bill tobacco dependence treatment under the primary disorder. Steps to change can be as simple as forbidding smoking on the premises of treatment facilities, not allowing staff members to smoke with patients, and changing the name of smoking breaks to simply “breaks.” (pp. 228–235)

## INTEGRATING TOBACCO DEPENDENCE TREATMENT AND TOBACCO-FREE STANDARDS INTO ADDICTION TREATMENT: NEW JERSEY’S EXPERIENCE

New Jersey was the first State to require that residential addiction treatment facilities assess and treat patients for tobacco dependence and maintain smoke-free grounds. In this article, Drs. Jonathan Foulds, Jill M. Williams, Bernice Order-Connors, Nancy Edwards, Martha Dwyer, Anna Kline, and Douglas M. Ziedonis review this policy change and its implementation and effects. The authors found that treatment for tobacco dependence can be successfully implemented in addiction treatment programs through policy regulation, training, and the integration of nicotine replacement therapy into treatment. The new regulations did not lead to patients leaving treatment early; in fact, two-thirds of smokers surveyed wanted to quit. Staff members were often resistant to the new policy change, and implementing smoke-free grounds was the most challenging aspect of the change.. The smoke-free policy was not strictly enforced, which the authors suggest may have compromised its effectiveness. (pp. 236–240)

